# Herbivory Modifies the Role of Spatial Processes in a Grassland Plant Metacommunity

**DOI:** 10.1111/ele.70257

**Published:** 2025-11-19

**Authors:** Lena Huovinen, Marjo Saastamoinen, Jonathan M. Chase, Anna‐Liisa Laine, Aleksi Räsänen, Anu Eskelinen

**Affiliations:** ^1^ Ecology and Genetics Unit University of Oulu Oulu Finland; ^2^ Faculty of Biological and Environmental Sciences University of Helsinki Helsinki Finland; ^3^ German Center for Integrative Biodiversity Research (iDiv) Leipzig Germany; ^4^ Geography Research Unit University of Oulu Oulu Finland

**Keywords:** biodiversity, habitat area, habitat connectivity, herbivory, metacommunity ecology

## Abstract

Trophic interactions can strongly influence metacommunity dynamics and patterns of biodiversity in spatially heterogeneous environments. Theory predicts that herbivory facilitates plant species coexistence at small scales by reducing extinctions and promoting colonisations but reduces diversity at larger scales by promoting dominance of herbivore‐resistant species. We examined how mammalian herbivory interacts with habitat size and connectivity to affect plant diversity in a unique, naturally fragmented grassland metacommunity system located in Southern Finland. We found that herbivory increased plant diversity across scales of measurement. In addition, herbivory reversed the diversity–area relationship such that there was a positive diversity–area relationship in grazed grasslands, but a negative relationship in ungrazed grasslands. Connectivity exhibited a unimodal relationship with diversity but did not interact with herbivory. Our empirical results demonstrate that herbivores can promote plant coexistence across scales and highlight the interplay between habitat area and trophic interactions in facilitating plant biodiversity in grassland metacommunities.

## Graphical abstract

We empirically examined how mammalian herbivory interacts with habitat size and connectivity to affect plant diversity in a natural grassland metacommunity. We found that herbivory increased plant diversity across scales of measurement and reversed the diversity–area relationship, showing a positive relationship in grazed and a negative relationship in ungrazed grasslands. Our results demonstrate the importance to incorporate trophic interactions into metacommunity theory and biodiversity predictions of spatially heterogeneous systems.

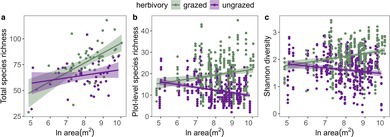

## 1 Introduction

Biodiversity is changing rapidly in the Anthropocene, and understanding and predicting these changes is a central challenge for ecology. This difficulty arises because biodiversity patterns are shaped by several interacting processes that operate across spatial scales, including dispersal, habitat configuration and biotic interactions (Leibold et al. 2004; Vellend 2017). The metacommunity framework integrates these spatial factors and local drivers; it provides a structured approach to assess their individual and combined effects on species distributions, community dynamics and biodiversity (Chase et al. 2020; Holyoak et al. 2005; Leibold and Chase 2018). Although spatial processes, that is, differences in habitat size and connectivity (Damschen et al. 2019; Germain et al. 2017; MacArthur and Wilson 1967), and local ecological interactions (Olff and Ritchie 1998; Tilman 1982; Zobel 1997) are each known to influence local community dynamics separately, the interplay between spatial and local biotic factors is rarely studied simultaneously (Chase et al. 2020; Holyoak et al. 2020). As a result, we lack a comprehensive understanding of their relative contributions, limiting our ability to generalise across systems and predict biodiversity responses to environmental change.

In metacommunities, large habitats can support higher species diversity due to sampling effects (i.e., with increasing grassland size more species out of the species pool are sampled), higher environmental variation that supports possibilities for niche differentiation and coexistence, and neutral effects (Connor and McCoy 1979; Leibold et al. 2004; MacArthur and Wilson 1967; Rosindell and Chisholm 2021). Further, well‐connected habitats can maintain higher species diversity as greater connectivity supports higher colonisation and lower extinction rates (Damschen et al. 2019; Holyoak et al. 2005; MacArthur and Wilson 1967). On the other hand, high connectivity and colonisation rates can also homogenise communities at both small and large scales, depending on whether they promote the colonisation of highly dominant competitors, potentially resulting in a unimodal diversity‐connectivity relationship (Kneitel and Miller 2003; Leibold and Chase 2018; Mouquet and Loreau 2002).

At a local scale, species interactions, including those within (i.e., competition) and between trophic levels (e.g., herbivory), interact with environmental conditions to structure assemblages and their diversity (Olff and Ritchie 1998; Tilman 1982; Zobel 1997). In terrestrial grassland communities, mammalian herbivory is a key factor that can strongly modify plant community composition and diversity (Collins et al. 1998; Huntly 1991; Olff and Ritchie 1998). These effects can be mediated through shifts in biomass, competitive interactions, and trade‐offs related to plant growth, competition and defence (Coley et al. 1985; Eskelinen et al. 2022; Jia et al. 2018; Lind et al. 2013). Although herbivore effects on plant diversity can vary, for example, depending on grazing intensity, soil nutrient availability, habitat productivity, and evolutionary history, exerting a negative impact on diversity in resource‐poor habitats, at high intensity, and in evolutionary young systems (Bakker et al. 2006; Price et al. 2022; Proulx and Mazumder 1998), generalist mammalian grazers are often found to promote grassland plant diversity (Collins et al. 1998; Borer et al. 2014; Eskelinen et al. 2022; Hillebrand et al. 2007; Olff and Ritchie 1998; Pringle et al. 2023). For example, herbivores can increase grassland diversity by consuming superior competitors for light (Borer et al. 2014; Eskelinen et al. 2022) and by reducing the accumulation of dead plant material, that is, litter, which can further maintain richness through enhancing recruitment from seed (Facelli and Pickett 1991; Jessen et al. 2023).

Incorporating trophic interactions, such as herbivory, into the metacommunity framework can fundamentally alter the outcome of metacommunity dynamics (Guzman et al. 2019; Holyoak et al. 2020; Leibold and Chase 2018). Herbivory could, for example, alter the relationship between habitat size and plant diversity in metacommunities (De Bello et al. 2007; Gravel et al. 2011). Herbivores can facilitate plant coexistence at small scales by reducing extinction and promoting colonization rates (Bakker and Olff 2003; Eskelinen et al. 2016, 2022). Large grazers can also enhance the spatial heterogeneity in vegetation structure (Olff et al. 1999; Trepel et al. 2024) which could promote higher plant diversity at larger scales (De Bello et al. 2007). However, if herbivores consistently consume grazing‐tolerant and/or palatable species, it could maintain plant diversity at small scales but diminish plant diversity at larger scales by promoting dominance of a restricted pool of grazing‐avoiding plant species (Olff and Ritchie 1998; Zhang et al. 2013).

Large herbivores could further alter metacommunity dynamics through their effects on plant dispersal. Herbivory can enhance the relative importance of dispersal for plant diversity by consuming biomass of competitively dominant species and creating small‐scale disturbance, thereby keeping communities more open for immigration (Bakker and Olff 2003; Eskelinen et al. 2016; Olff and Ritchie 1998). Herbivores can also transport seeds through their dung and with their fur (Jordano 2017). Since dispersal is inherently linked to habitat isolation (Vandvik and Goldberg 2006), connectivity and herbivory could interact; in the presence of herbivores, connectivity and diversity could show a positive association, while in the absence of herbivores, this relationship could be weaker because of a stronger role of competition driving plant community diversity at small scales (Chase et al. 2010; Leibold and Chase 2018; Tilman 1994). Furthermore, plant functional traits related to dispersal in space, resource use, and defence could provide an important tool to explain potential relationships and mechanisms between the spatial context, grazing and diversity (Díaz et al. 2007; Funk et al. 2017; Leibold and Chase 2018). However, despite multiple mechanisms by which herbivory can alter plant metacommunity dynamics, real‐world empirical studies addressing interactions among herbivory, habitat size and connectivity and traits related to these relationships, are scant.

**FIGURE 1:**
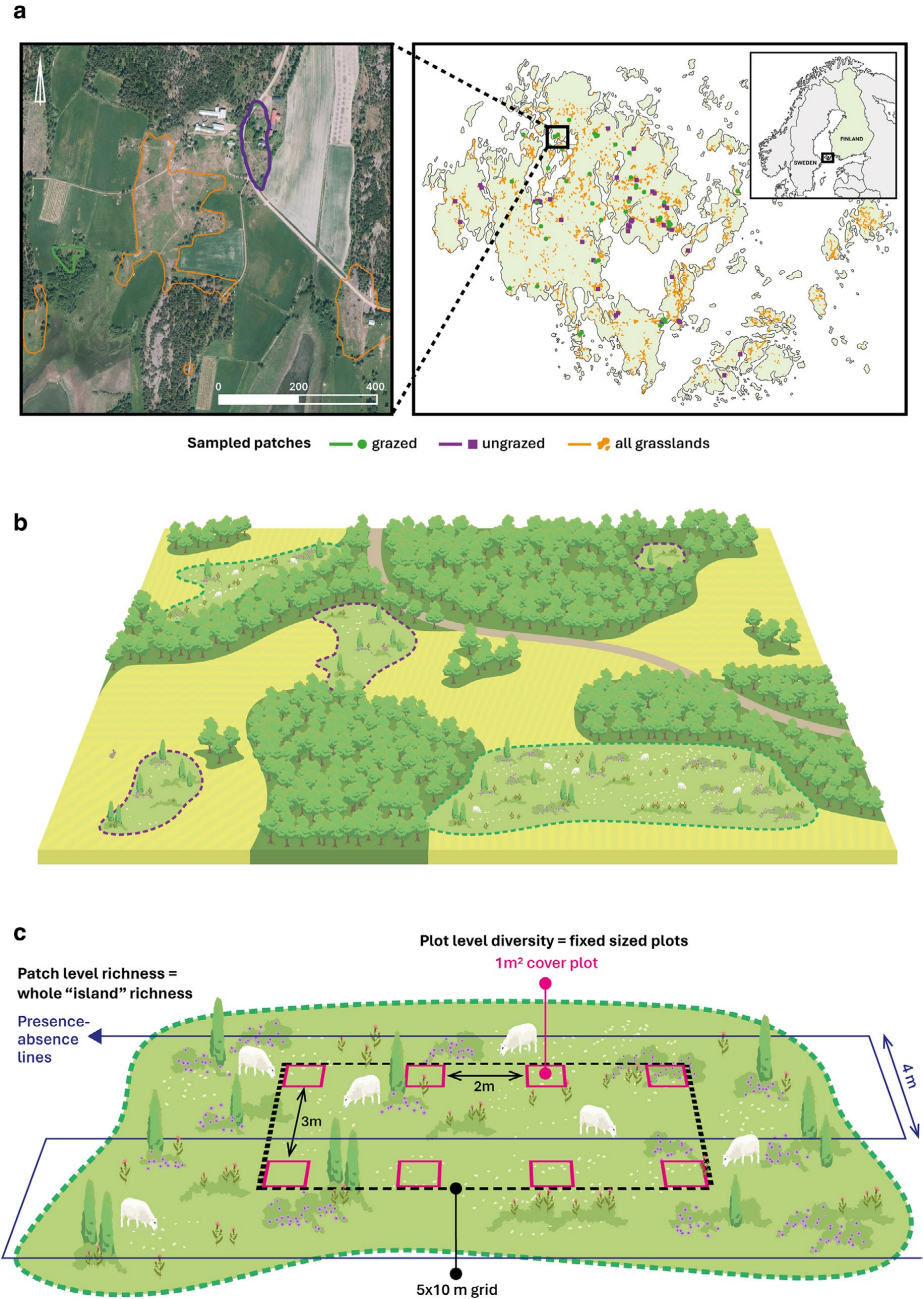
(a) On the right: A map of the study system in the Åland archipelago in South‐Western Finland, which consists of over 4000 grassland patches. We sampled 90 grassland patches that represented a range of sizes (large and small) and connectivity (well‐connected and isolated), and of which 46 were grazed and 44 were ungrazed. On the left: An example of the patches in the landscape (background: Aerial image by National Land Survey of Finland, taken in 2022). (b) An illustration of a fragmented grassland plant metacommunity system with discrete grassland patches that are surrounded by a matrix of forest or agricultural fields, or a mixture of both, and were never surrounded by water (are therefore not true ‘islands’). All grassland patches had continuous vegetation cover, were not excessively rocky or dry, were not visibly disturbed by humans, and did not include human‐introduced forage or other cultivated, non‐native species. (c) Sampling design of plant community composition and diversity in the 90 grassland patches. Within each grassland patch, we established a 5 × 10 m grid consisting of eight fixed sized 1 m^2^ plots arranged in two lines that were three meters apart. The grid was placed approximately in the middle of each grassland. Additionally, we recorded the presence/absence of all species not present in the grid along transects (on both sides approximately up to 1 m distance from the transect) that were four meters apart parallel to the long side of the grid. The transects covered the whole grassland area.

**FIGURE 2:**
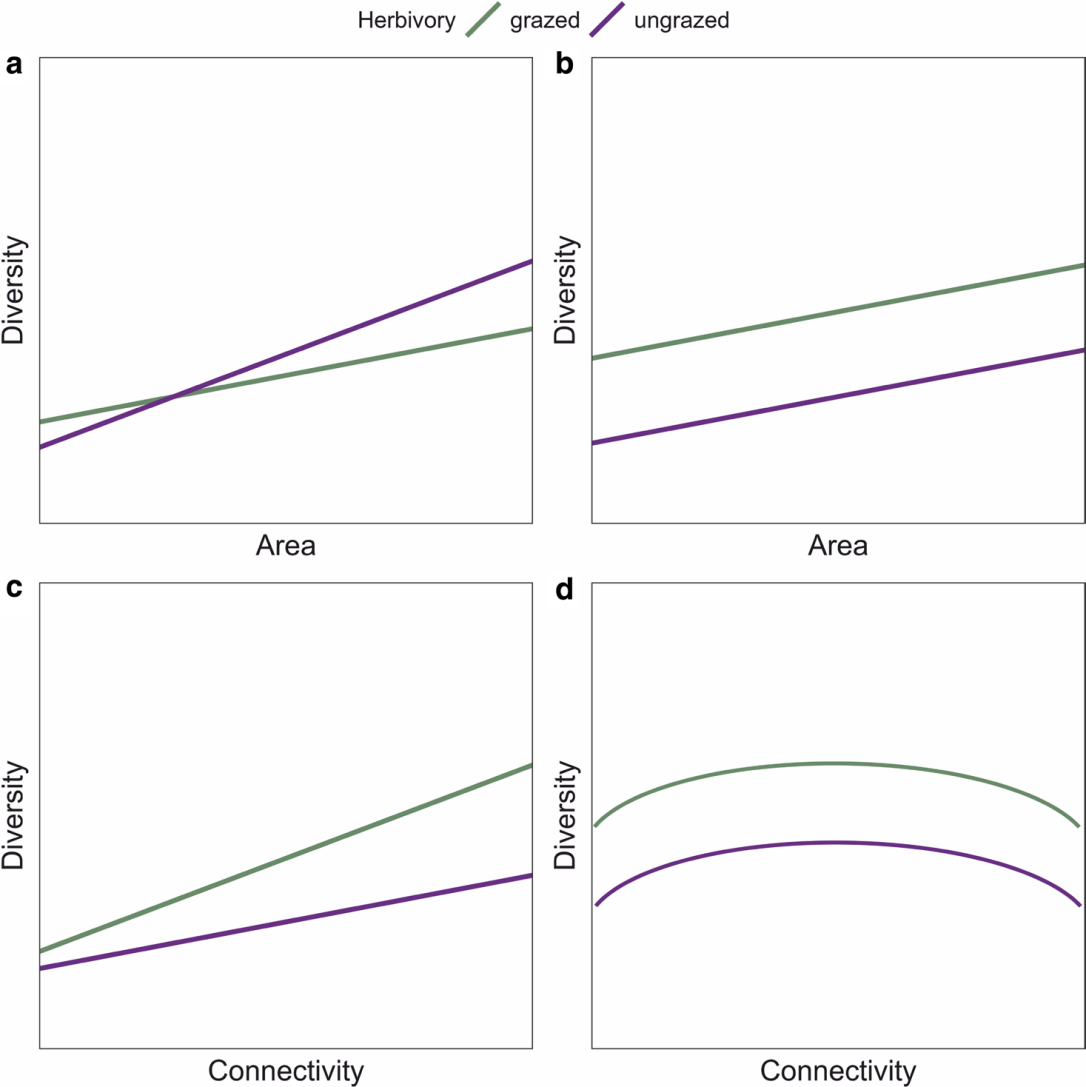
Predictions for plant diversity as a function of herbivory and habitat area (a, b) and herbivory and connectivity (c, d). (a) Herbivores can increase diversity at small scales (i.e., in small areas) but lead to homogenization and decreased diversity at larger scales (i.e., in larger areas) or, alternatively, (b) herbivores can promote plant diversity independently of the scale/area. Herbivore effects on diversity can further depend on habitat connectivity such that (c) high connectivity is associated with high diversity only in the presence of herbivores or, alternatively, (d) herbivory promotes diversity independently of connectivity, which shows a unimodal relationship with diversity.

Here, we examined the joint effects of spatial variation (habitat size and connectivity) and mammalian herbivory on plant diversity in a naturally fragmented grassland system on the Åland archipelago of Southwest Finland (Figure 1a,b). This system has served as a model system for modern metapopulation theory and has provided critical knowledge of how species persist in fragmented landscapes with varying habitat patch size and connectivity (Hanski 1999; Jousimo et al. 2014). We sampled plant diversity in 90 discrete grassland patches that varied in isolation and size, and approximately half of them were grazed and half were ungrazed by domestic mammals. We did our sampling on two scales, both across grasslands of distinct size (i.e., ‘islands’) and in fixed‐sized plots within different‐sized grasslands (Figure 1c), as these distinct sampling scales can reveal metacommunity processes and other mechanisms acting behind the diversity–area–herbivory relationship (Chase et al. 2019; Gooriah et al. 2021). We hypothesized that (i) grazing has a positive effect on plant diversity at small scales (i.e., in small areas); however, this positive impact disappears at larger scales (i.e., in larger areas) due to increased dominance of grazing‐avoiding species (Figure 2a). Alternatively, (ii) herbivory promotes higher plant diversity irrespective of the scale (i.e., in smaller and larger areas) (Figure 2b). We further predicted that (iii) connectivity and herbivory interact in such a way that high connectivity is linked to high diversity more strongly in the presence of herbivores (Figure 2c) or, alternatively, (iv) herbivory promotes diversity independently of connectivity, which shows a unimodal relationship with diversity (Figure 2d). We further used community‐weighted mean (CWM; Lavorel et al. 2008) traits to analyze functional trait responses to area, connectivity and herbivory.

## 2 Methods

### 2.1 Study System

Our study was conducted in the Åland Islands archipelago in Southern Finland. This archipelago spans over 1554 km^2^ of landmass comprising a 689 km^2^ main island, several medium‐sized islands and many small islands. The highest point is 129 m a.s.l. (Statistics Finland 2023). The mean annual temperature between 1991 and 2020 is 6.7°C and the mean annual precipitation is 586 mm (Finnish Meteorological Institute 2024). The Åland Islands study system consists of over 4000 grassland habitat patches and has served as the metapopulation model system for the butterfly *Melitaea cinxia (L*.) (Hanski 1999; Ojanen et al. 2013) and *Plantago lanceolata (L.)* pathogens (Jousimo et al. 2014). The grasslands of varying sizes are distributed over the main island and many smaller islands at varying distances from each other, and are surrounded by a matrix of agricultural fields or forests. Approximately 40% of these patches are grazed by sheep, cattle or horses (Ojanen et al. 2013). In 2022, we chose 90 grassland patches that represented a range of sizes (small to large) and connectivity (well connected to isolated), of which 46 were grazed and 44 were ungrazed (Figure 1). In general, our study landscape is characterised by a fine‐grained mosaic of grazed and ungrazed patches that are often located within tens of meters of each other and occur on the same underlying habitat matrix. We carefully chose the patches to represent all combinations of connectivity, size and herbivory (e.g., small and larger grazed and ungrazed patches). We preselected the patches using the Earthcape database (Meyke 2019), maintained by the University of Helsinki for *M. cinxia* butterfly and *P. lanceolata* pathogen studies (Ojanen et al. 2013). We further visited the patches in person to check for their suitability for this study prior to data collection. We only used grassland patches that had spatially continuous vegetation cover (i.e., were not excessively rocky), were treeless and not visibly disturbed by humans. Further, we did not include pastures with human‐introduced sown species or transitioning grassland‐forest vegetation. We defined an ungrazed patch as a patch that had not been grazed since at least 2016, except for one patch that had been grazed until 2019. Grazed patches had been continuously grazed for many decades and varied in the intensity of grazing, some being heavily and some only lightly grazed and many were rotationally grazed (L. Huovinen and A. Eskelinen, personal observation). A more detailed description of patch properties is provided in the Supporting Information.

During plant community sampling in 2022 and 2023 (see below), we recorded grassland patch areas by logging GPS points in the field and then calculated the extent of the areas in ArcGIS version 10.8.1 (ESRI 2024). The patches varied in size from 118 to 23,004 m^2^ with an average size of 5271 m^2^. To confirm that all potentially suitable grassland area within a 1 km radius around the selected 90 grassland patches was included in our connectivity measure, we first visually interpreted aerial images around each focal patch for suitable patches that were missing in the Earthcape database. Second, we visited all these potentially suitable patches in the field to validate their suitability, recorded their areal extent as above, and added them to our database.

We calculated habitat connectivity as a modified version of a ‘buffer measure’ described in Moilanen and Nieminen (2002). Our modified connectivity measure considers both the dispersal distance between the focal patch and adjacent patches and the area of adjacent patches within a buffer around the focal patch.

$$\text{Connectivity}=\sum_{\begin{array}{c} j\neq i\\ d_{i,j}<r \end{array}}A_{j}\times \left(r-d_{i,j}\right)$$

where *r* = maximum dispersal distance here defined as 1 km, *d*_*i,j*_ = the distance between sampled focal patch *i* and adjacent patch *j*. Only distances *d*_*i,j*_ < *r* were considered. *A*_*j*_ equals the area of the adjacent patch. Distance was measured from edge to edge. The distance *d*_*i,j*_ was calculated in ArcGIS with the ‘generate near table’ tool and it was scaled to the maximum dispersal distance *r* with *r‐d*_*i,j*_. We chose 1 km as the maximum dispersal distance since this is sufficiently greater than the maximum distance of different dispersal distance models for herbaceous plants; probabilities of dispersal beyond a few hundred meters in plants are extremely low (Bullock et al. 2002; Nathan 2006; Tamme et al. 2014). In general, connectivity measures utilising negative exponential dispersal kernels (Moilanen and Nieminen 2002) are based on average dispersal distance for a single species. As average dispersal distances vary heavily between species and communities, choosing a α parameter for a plant community is not necessarily meaningful. However, we also tried a negative exponential dispersal kernel connectivity measure (Moilanen and Nieminen 2002, 1/alpha = average dispersal distance), with the average ‘community’ dispersal distance 400 m (alpha = 0.0025) based on Lososová et al. (2023), where the dispersal distance (animal dispersal) was 400 m for 50% of the measured distances. These two connectivity measures were highly correlated (Pearson *r* = 0.96, *p* < 0.0001) and produced qualitatively similar results; data and results are therefore not shown. Additionally, we calculated the distance to the nearest patch or each focal patch using ArcGIS, the distance calculated from edge to edge of two adjacent patches. The distance to the nearest patch ranged between 14 and 546 m.

### 2.2 Plant Community Sampling

We collected plant community data from the chosen 90 grassland patches from mid‐June to the end of July (peak biomass) in 2022 and 2023. Different grassland patches were sampled in different years; plots were therefore surveyed only once in one of the 2 years. In each grassland patch, we placed a grid of 5 × 10 m approximately in the middle of the patch, with a few exceptions due to difficult terrain. The grid consisted of eight 1 m^2^ plots in two rows that were three meters apart (Figure 1). At each grassland patch, we visually estimated cover for all vascular plant species occurring in the 1 m^2^ plots. In total, we sampled 720 1 m^2^ plots for plant communities. Because two people collected plant community data, we calibrated the sampling by frequently estimating covers together to ensure similar cover estimates. We recorded vascular plant presence/absence outside the grid along transects that were parallel to the long side of the grid, spaced four meters apart and spanning the area of the whole patch. Larger patches had therefore longer and more transects.

We calculated species richness as the number of vascular plant species in each 1 m^2^ plot. We quantified the Shannon‐Wiener index as *H* = −∑_*i*_ *p*_*i*_log_1_ *p*_*i*_, where *p*_*i*_ is the proportional abundance of species (Hurlbert 1971). Evenness was calculated as *H*/log(richness) (Pielou 1966). We calculated Shannon diversity and evenness using the ‘vegan’ package (Oksanen et al. 2024). We also calculated whole patch level richness as the number of vascular plant species summed across the plots and transects within each patch.

### 2.3 Trait Measurements

We used functional traits linked to herbivory, resource use, competition for light and dispersal in space. We collected data on plant height (from the ground to the highest photosynthetically active part of the plant in cm) as plant height can affect susceptibility to grazing and ability to compete for light (Craine and Dybzinski 2013; Díaz et al. 2007). We further measured specific leaf area (SLA; cm^2^ g^−1^; area of a fresh leaf divided by its oven‐dried mass) as it is connected to plant growth (Wright et al. 2004), and foliar condensed tannin concentrations (mg g^−1^) and foliar carbon to nitrogen ratio (C:N; from C, N %), which both relate to herbivory defence and plant growth (Coley et al. 1985; Hanley et al. 2007). We measured water‐use efficiency (WUE; measured as the proportion of the isotopes ^13^C/^12^C compared to the VPDB standard, where less negative values equal higher WUE; Farquhar and Wong 1984) that relates to drought tolerance (Moreno‐Gutiérrez et al. 2012). All traits were collected from 5 to 10 individuals (depending on the trait) following standard protocols (Pérez‐Harguindeguy et al. 2016). The traits were collected in the summers 2022–2024 from the same grasslands where plant community sampling occurred (but outside the plots) or from nearby grasslands. Our trait data represented more than 90% of the cover of all species occurring in the plots. Additionally, we obtained data on dispersal potential from Lososová et al. (2023), which is a measure on a scale of 1–6 that estimates potential dispersal distances based on dispersal mode, seed release height, morphology of dispersal units, plant height, life form, seed mass and habitat. (scale: 1 = 0.1–1 m, 2 = 1–5 m, 3 = 2–15 m, 4 = 40–150 m, 5 = 10–500 m, 6 = 400–1500 m). We calculated CWM traits (Lavorel et al. 2008) for each trait per plot as the relative abundance of the species (% cover) multiplied by the species' trait values and summed across species. We used the ‘FD’ package in R (Laliberté et al. 2024) to calculate CWM trait values. The detailed description of trait measurements is provided in Supporting Information.

### 2.4 Statistical Analyses

We used statistical software R version 4.4.1 (R Core Team 2024) for all analyses. To test how herbivory, habitat size, connectivity, and their interactions affect plant species richness, diversity and evenness, we used linear mixed effect models (LME; Pinheiro and Bates 2000; ‘lme’ function in the ‘nlme’ package in R, Pinheiro et al. 2024) where plot‐level species richness, Shannon diversity, and evenness, were response variables, each in their own model, and patch size, either connectivity or distance to nearest patch, grazing and the interactions between grazing and patch size and grazing and connectivity/distance to nearest patch were fixed factors. Distance to nearest patch and connectivity were not analysed in the same models as they were heavily correlated. We used similar models to test the effects of herbivory, habitat size and connectivity on CMW functional traits (height, SLA, tannins, C:N, WUE and dispersal potential). We included patch identity as a random factor in the models to consider that plots were nested within the patch, and due to spatial autocorrelation of patches (i.e., that some patches were closer to each other; Figure 1a) in our sampling.

We used Generalised Least Squares (GLS; ‘gls’ function in the ‘nlme’ package in R, Pinheiro et al. 2024) models without random structure to test how the same predictor variables as above were related to whole grassland level richness. We used GLS instead of a simple linear model to include a spatial autocorrelation structure to account for the fact that some patches were closer to each other in our sampling system (Figure 1a). We fitted the spatial autocorrelation as an exponential correlation structure with the coordinates of the centre points of the habitat patches. We also ran similar simple linear models without a spatial autocorrelation structure and found that the results were qualitatively similar (not shown), showing that considering the spatial autocorrelation did not affect our results.

For all models including distance to the nearest patch, we removed one patch that was at a significantly greater distance than all others. We transformed area with natural logarithms in all models (Gleason 1922) and added a second‐order polynomial function to fit distance to the nearest patch/connectivity for models with richness and Shannon diversity. Model fit was inspected using model diagnostic plots and response variables were transformed if needed to meet model assumptions (see Tables S5–S8).

## 3 Results

We recorded a total of 238 vascular plant species in the 1 m^2^ plots, with species richness per plot varying between 2 and 45. The total number of plant species in the grassland patches, including species that we found along the transects, varied between 29 and 118.

**FIGURE 3:**
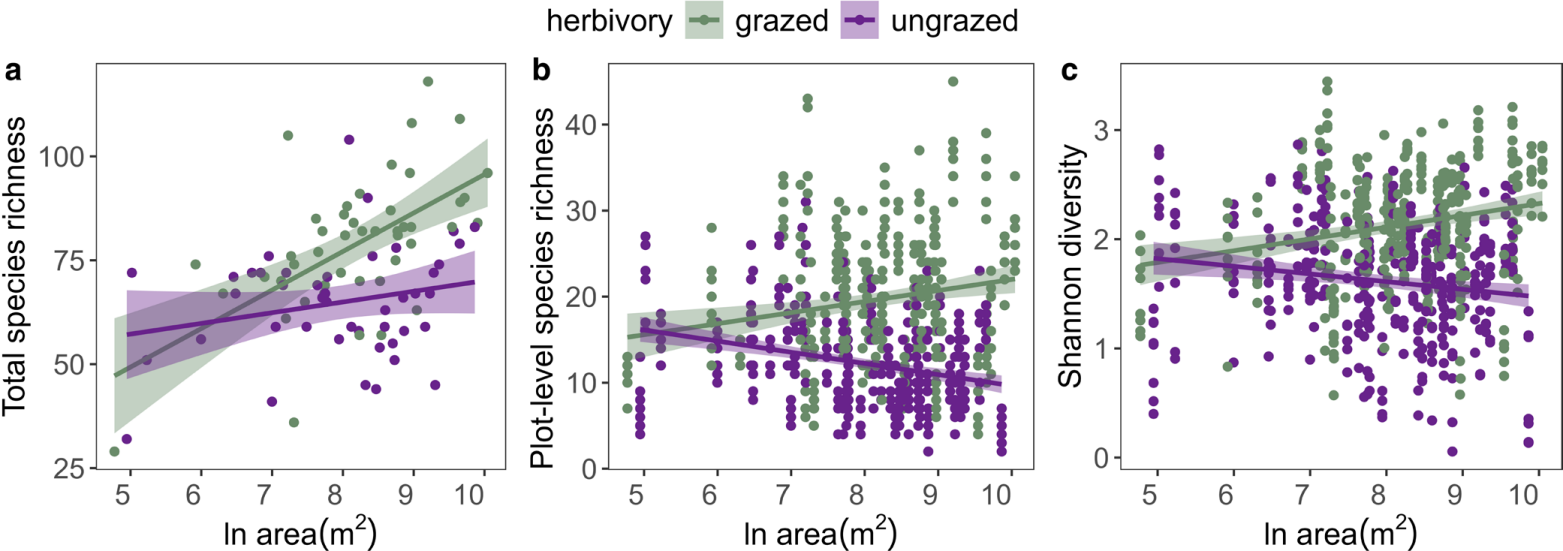
Plant species richness and Shannon diversity in relation to habitat size in grazed (green) and ungrazed (purple) grasslands. (a) Total plant species richness, measured as a sum of species occurring along transects covering the whole patch area and species found in eight 1 m^2^ plots in the middle of each patch, in relation to ln area (m^2^) and herbivory (*R* ^2^_nagelkerke_ = 0.43, *P*_area_ < 0.0001, *P*_herbivory_ < 0.0001, *P*_herbivory × area_ = 0.0441); (b) plant species richness in fixed sized 1 m^2^ plots (eight replicates per grassland) in relation to ln patch area (m^2^) and herbivory (*R* ^2^_conditional_ = 0.81, *P*_herbivory_ < 0.0001, *P*_herbivory × area_ = 0.0209); (c) Shannon diversity in fixed sized 1 m^2^ plots (eight replicates per patch) in relation to ln patch area (m^2^) and herbivory (*R* ^2^_conditional_ = 0.68, *P*_herbivory_ < 0.0001, *P*_herbivory × area_ = 0.0201).

**FIGURE 4:**
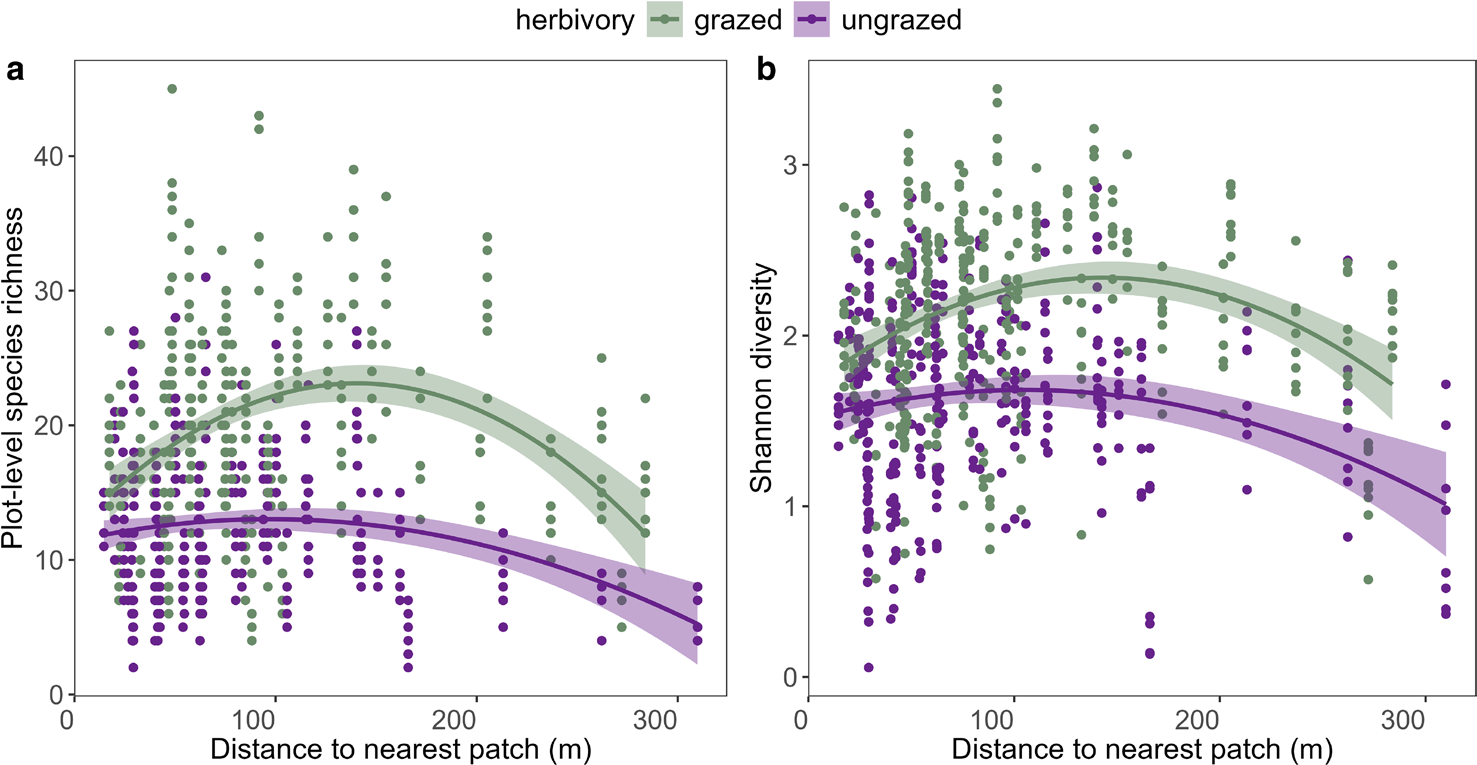
(a) The relationship between plant species richness and distance to the nearest patch (m) (*R* ^2^_conditional_ = 0.81, *P*_herbivory_ < 0.0001, *P*_distance to the nearest patch_ = 0.0050) and (b) the relationship between Shannon diversity and distance to the nearest patch (m) (*R* ^2^_conditional_ = 0.68, *P*_herbivory_ < 0.0001, *P*_distance to the nearest patch_ = 0.0080) in fixed‐sized 1 m^2^ plots (eight replicates per patch).

In general, models with distance to nearest patch and connectivity as predictors produced qualitatively similar results and, for simplicity, we only report results from models with distance to nearest patch as a predictor here. Model results with connectivity as a predictor are reported in Tables S6 and S8. We found that both whole patch level species richness and plot‐level species richness and diversity were significantly positively affected by herbivory (*F*_1,81_ = 34.8, *p* < 0.0001; *F*_1,81_ = 38.0, *p* < 0.0001; *F*_1,81_ = 36.6, *p* < 0.0001, respectively, Table S5, Figure 3a–c). Herbivory also interacted with habitat area on whole patch and plot levels. At whole patch level, area was positively related to richness in both ungrazed and grazed grasslands and the positive impact of herbivory on richness was greater in larger grasslands (*F*_1,81_ = 4.2, *p* = 0.0441, Table S5, Figure 3a). In contrast, at plot level (i.e., in fixed sized plots), herbivory reversed the richness‐area relationship such that there was a positive association between richness and area in grazed grasslands but a negative association in ungrazed grasslands (*F*_1,81_ = 5.6, *p* = 0.0209, Table S5, Figure 3b). We found the same interaction for Shannon diversity (*F*_1,81_ = 5.6, *p* = 0.0201, Table S5, Figure 3c). Plot level richness and Shannon diversity showed a unimodal relationship with both distance to the nearest patch and connectivity, such that richness and diversity both peaked at intermediate distance to the nearest patch (*F*_1,81_ = 8.3, *p* = 0.0050; *F*_1,81_ = 7.4, *p* = 0.0080, respectively, Table S5, Figure 4a,b) and at intermediate connectivity (*F*_1,84_ = 5.3, *p* = 0.0051; *F*_1,84_ = 3.1, *p* = 0.0475, respectively, Table S6, Figure S1a,b). Evenness increased with herbivory but was not influenced by any other explanatory variables (*F*_1,81_ = 17.2, *p* = 0.0001, Figure S2, Tables S5 and S6).

**FIGURE 5:**
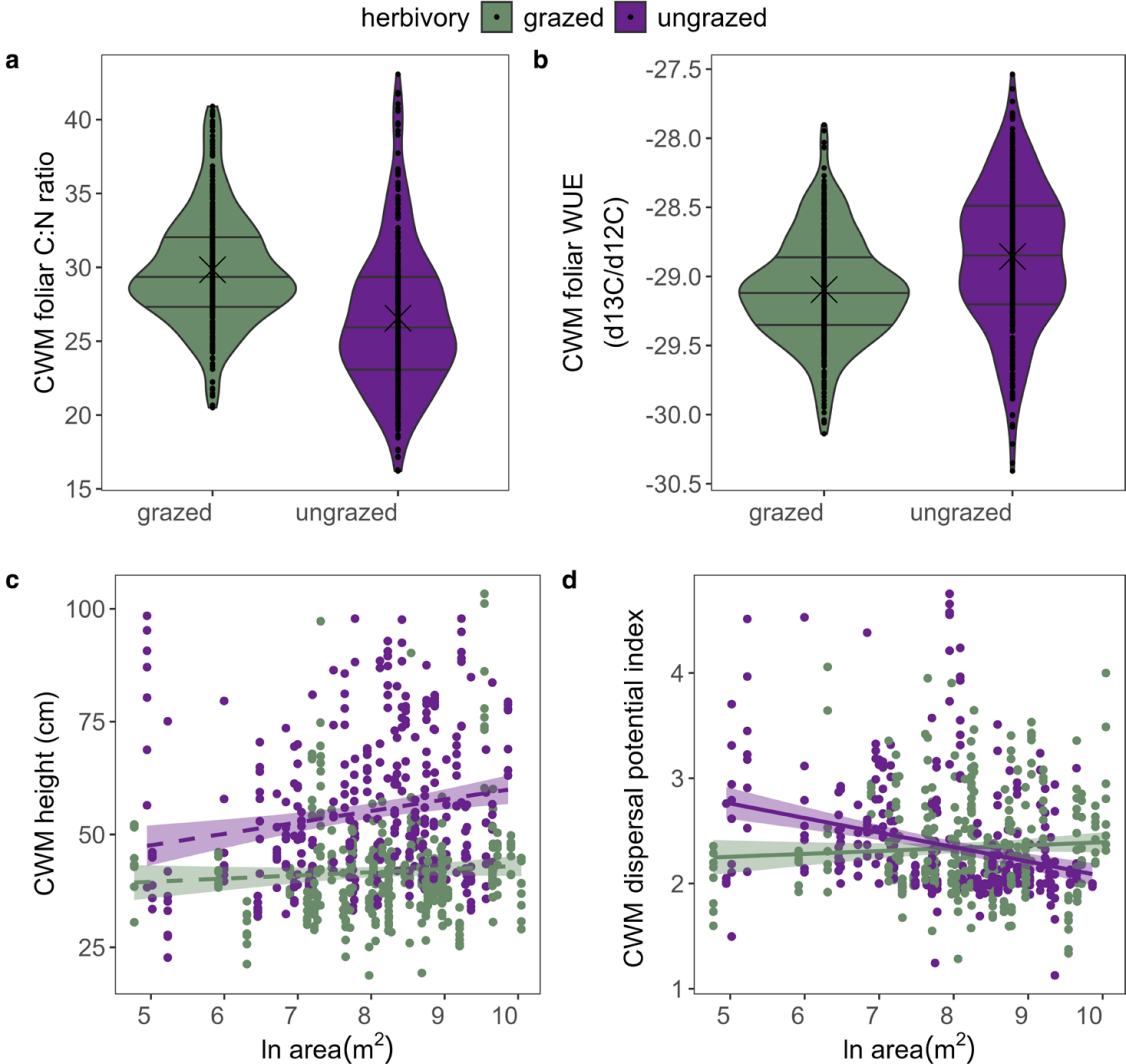
CWM traits in relation to herbivory and habitat size. (a) CWM C:N (%) in grazed and ungrazed grasslands (*R* ^2^_conditional_ = 0.54, *P*_herbivory_ < 0.0001); (b) CWM WUE (water use efficiency,13C/12C isotopes compared to the VPDB standard) in grazed and ungrazed grasslands (*R* ^2^_conditional_ = 0.43, *P*_herbivory_ = 0.0002); (c) plant height (cm) in relation to ln habitat size in grazed and ungrazed grasslands (*R* ^2^_conditional_ = 0.68, *P*_area_ = 0.0778, *P*_herbivory_ < 0.0001); (d) dispersal potential (scale: 1 = 0.1–1 m, 2 = 1–5 m, 3 = 2–15 m, 4 = 40–150 m, 5 = 10–500 m, 6 = 400–1500 m) in relation to ln habitat size in grazed and ungrazed grasslands (*R* ^2^_conditional_ = 0.51, *P*_area_ = 0.0490, *P*_herbivory × area_ = 0.0113). Dashed fitted line indicates marginally significant effect.

Herbivory significantly affected the CWM of plant height, C:N and WUE, such that grazed grasslands were more dominated by shorter species that had a high C:N ratio and low WUE (*F*_1,83_ = 34.4, *p* < 0.0001; *F*_1,83_ = 25.3, *p* < 0.0001; *F*_1,83_ = 15.8, *p* = 0.0002, respectively; Figure 5a,b, Table S7). The CWM of height was weakly positively associated with patch area, especially in ungrazed grasslands, although this relationship was not quite statistically significant (*F*_1,83_ = 3.2, *p* = 0.0778, Figure 5c, Table S7). Further, herbivory significantly interacted with patch area, such that the CWM of dispersal potential was negatively related to area in ungrazed grasslands but not in grazed grasslands (*F*_1,83_ = 6.7, *p* = 0.0113, Figure 5d, Table S7). In other words, large and grazed grasslands exhibited a higher CWM of dispersal potential than large ungrazed patches, while small and grazed grasslands showed a lower CWM of dispersal potential than small ungrazed patches. The CWM of SLA and tannin showed no significant responses to any of the explanatory variables (results not shown).

## 4 Discussion

We used a real‐world grassland metacommunity, an iconic model system for modern metapopulation theory (Hanski 1999; Jousimo et al. 2014), to investigate how the spatial context (habitat size and connectivity) and mammalian herbivory jointly affect plant biodiversity. We show that while herbivory by large mammalian grazers generally increased plant diversity across scales, it also modified the effect of habitat size on diversity. In the presence of herbivores, larger grasslands supported greater diversity (i.e., there was a positive species‐area relationship), but in the absence of herbivores, larger grasslands supported lower diversity. While this relationship was true both at the whole grassland level (i.e., across grassland islands) and in fixed‐sized plots, its slope varied. We also demonstrate that the highest plant diversity was observed at intermediate habitat connectivity, but this effect was not modified by herbivory. These findings highlight the interplay between spatial context and trophic interactions in facilitating species coexistence in grassland plant metacommunities.

Opposite to previous theoretical work predicting that herbivory could negatively affect plant diversity at larger scales by favouring the dominance of grazing‐avoiding species (Olff and Ritchie 1998, see also; Zhang et al. 2013), our results demonstrate that herbivory promotes diversity both across grasslands of distinct size (i.e., ‘islands’) and in fixed‐sized plots within different‐sized grasslands which, to our knowledge, have not been shown in any previous studies. In grassland ecosystems that are not strongly limited by nutrients and water, and are dominated by palatable graminoids and forbs, generalist grazers are not likely to impose strong selection for grazing avoidance (i.e., plants being unpalatable to herbivores) (Olff and Ritchie 1998). Rather, grazing in such systems operates through removing biomass of competitively dominant, palatable species, and alleviates competition for light (Borer et al. 2014; Eskelinen et al. 2022; Koerner et al. 2018), which should not reduce the size of the local species pool. However, these outcomes could differ, for example, if heavy grazing is coupled with extreme aridity or nutrient scarcity, if herbivores target subordinate forbs, and in evolutionary young grazing systems (Milchunas et al. 1988; Price et al. 2022; Proulx and Mazumder 1998). Further, herbivory can support more heterogeneous vegetation and environmental conditions by creating variation in litter cover, bare ground and biomass (Trepel et al. 2024), which maintains niche dimensionality and allows more species to specialise under these distinct conditions. These could all benefit species' coexistence across scales.

While theoretical work suggests that trophic interactions, such as herbivory, might modulate species‐area relationships (Gravel et al. 2011; Ryberg and Chase 2007; Ryser et al. 2024), these relationships have rarely been empirically tested in terrestrial plant communities (but see Chaneton and Facelli 1991; De Bello et al. 2007). In our study, sampling both at whole grassland patch and fixed plot levels allowed us to investigate the processes that mediate herbivore effects on the richness‐area relationship (Chase et al. 2019; Gooriah et al. 2021). We found that, at the whole grassland level, there was a positive richness‐area relationship both in grazed and ungrazed grassland patches, and the slope of this relationship was steeper in grazed grasslands (i.e., the positive impact of herbivory on richness was greater in larger grasslands). In contrast, in our fixed sized (1 m^2^) plots, herbivory reversed the richness‐area relationship; we found a positive richness‐area relationship in grazed grasslands but a negative relationship in ungrazed grasslands. Several metacommunity processes and other mechanisms can contribute to explaining these findings.

First, both passive sampling (i.e., with increasing grassland size more species out of the species pool are sampled) and heterogeneity effects (i.e., larger grasslands can include more variable environmental conditions) (Chase et al. 2019; Connor and McCoy 1979; MacArthur and Wilson 1967) can contribute to explaining why we found a positive richness‐area relationship in ungrazed grasslands only when the whole grassland islands were considered. In general, heterogeneity effects could also explain diversity patterns in fixed‐sized plots (Chase et al. 2019), however, in our grassland system and with our fixed plot size (1 m^2^), heterogeneity effects likely play a greater role at larger scales. Our result that the positive richness‐area relationship was amplified in grazed grasslands suggests that herbivory can amplify either passive sampling or heterogeneity effects, or both, through promoting a greater local species pool and through creating greater spatial heterogeneity in vegetation and/or environmental conditions.

Second, our finding that, in fixed sized plots, the richness‐area relationship was strongly positive in the presence of grazers but negative in the absence of grazers, suggests that herbivory is a key factor that can drive disproportionate metacommunity effects (also called ‘area per se’ effects; Chase et al. 2019). Larger grasslands can maintain a greater number of species because they support more species per unit area, called positive disproportionate effects; if a positive richness‐area relationship is observed at the fixed sampling scale, disproportionate effects can drive diversity patterns (Chase et al. 2019; Leibold and Chase 2018). These positive disproportionate effects could arise, for example, if larger habitats support more colonisations and fewer extinctions per fixed area (Chase et al. 2019; MacArthur and Wilson 1967). In general, grazing mammals have been shown to facilitate species coexistence at small scales by alleviating competition for light (Borer et al. 2014; Eskelinen et al. 2022), therefore reducing extinction rates. They can also create small‐scale disturbances and reduce seed germination barriers, therefore increasing colonisation rates (Auffret and Cousins 2013; Bakker and Olff 2003; Eskelinen et al. 2016; Jessen et al. 2023). Our results suggest that these positive effects of herbivory on diversity through colonisation‐extinction dynamics also magnify with grassland size. If the species pool in large, grazed grasslands is greater than in small, grazed grasslands, as our results imply, then there should be more species available for colonisations and that could magnify the positive effects of grazing on fixed‐sized plots.

Third, in the absence of grazing, we found a negative relationship between diversity/richness and area in fixed‐sized plots, implying that in larger grasslands the absence of herbivory disproportionately negatively affected small‐scale species coexistence (more than in small grasslands). Importantly, these effects only occurred on a 1 m^2^ plot scale, while large, ungrazed grasslands still supported higher species richness on the total patch scale, implying that species interactions drive these effects. Larger grasslands may possess a greater chance than small grasslands to include a superior competitor for light through sampling effects (Chase et al. 2019; MacArthur and Wilson 1967) and through greater heterogeneity in environmental conditions (e.g., soil nutrients and moisture), that can support populations of resource‐demanding, highly competitive species. Further, due to positive biodiversity‐ecosystem functioning relationships (BEF; Hector et al. 1999; Tilman 2001), we could also expect that larger habitats, supporting more species, could also support higher productivity (Leibold et al. 2017; Rychtecká et al. 2014). These together with reduced ecological drift and more stable communities in large grasslands (Orrock and Watling 2010; Vellend 2017) can select for highly productive and competitive species, increase chances for competitive exclusion and lead to decreased diversity in fixed‐sized plots in the absence of herbivores.

Our nearly significant finding that large grasslands in the absence of herbivores were more dominated by tall plants (i.e., exhibited greater CWM height), than small grasslands, supports the above interpretation. In grassland ecosystems, plant height is generally expected to correlate with competitive ability for light (Craine and Dybzinski 2013; Reich 2014). Dominance by taller plants was further negatively related to the dominance of plants with lower dispersal ability, as predicted by the competition‐colonisation trade‐off (Tilman 1994). Besides these, several other metaecosystem or metacommunity processes could contribute to our finding. For example, in ungrazed small grasslands, diversity could be more heavily impacted by edge effects that could counteract the negative impact of the absence of grazing on diversity in fixed‐sized plots. Moreover, although we did not find a difference in total soil nitrogen and moisture in relation to grassland size (Table S1), soil processes and ecosystem functions at the metaecosystem level could link to these observed patterns in complex and generally little‐explored ways (Loreau et al. 2003); however, studying these is beyond the scope of our study. Finally, while we do not have detailed long‐term history of our grassland sites and do not have information related to how landowners originally chose these patches, most large ungrazed grasslands were grazed at some point during the past 30 years (Table S2), suggesting that cessation of grazing likely drives these patterns. Taken together, these results highlight that changes in extinction and colonisation rates imposed by herbivores can be fundamentally important for metacommunity processes and even reverse the observed diversity‐area relationship.

We observed a unimodal relationship between diversity and distance to nearest patch/connectivity in fixed sized plots, showing that close proximity to other grassland patches decreases diversity, and implying that high dispersal can homogenise communities (Cadotte 2006; Kneitel and Miller 2003; Mouquet and Loreau 2002; Vanschoenwinkel et al. 2013). Herbivory can keep local communities open for immigration from surrounding habitats (Olff and Ritchie 1998; Bakker and Olff 2003; Eskelinen et al. 2016) and, in our data, maintained higher diversity at all connectivity levels. In general, we would expect to observe an herbivore‐modulated response to connectivity only if herbivory alters extinction rates of dispersing species disproportionally at different connectivity values (Kneitel and Miller 2003) which, in our case, does not seem to happen. Herbivory could still affect wind‐ and animal‐dispersed species in different ways and increase the dispersal of animal‐dispersed species by acting as a dispersal vector (Auffret and Cousins 2013). Nevertheless, our findings suggest that the highest plant diversity can be achieved at intermediate connectivity levels in the presence of herbivores.

To conclude, our study highlights the importance of integrating trophic interactions into metacommunity theory and demonstrates that herbivory can be a key factor in modulating species‐area relationships. As a novel finding, our results suggest that grazing in grassland communities maintains diversity by altering different metacommunity processes underlying the diversity‐area relationship, such as habitat heterogeneity and disproportionate effects (Chase et al. 2019; Gooriah et al. 2021). Future experimental research should further disentangle and identify which of these components are actively modified by herbivory. While other recent studies emphasize the role of herbivores in protecting native plant diversity in invaded systems (Mungi et al. 2023), controlling the carbon cycle (Schmitz et al. 2023) and mitigating the loss of plant biodiversity under global environmental and climatic change (Kaarlejärvi et al. 2017; Post et al. 2023), our results highlight the fundamental importance of incorporating herbivory into biodiversity predictions of spatially heterogeneous and/or fragmented systems. In the absence of herbivory, we are likely to encounter relatively greater losses of plant biodiversity in large areas, which will have important ramifications for the conservation and restoration of grassland plant biodiversity.

## Author Contributions

Anu Eskelinen conceptualised and designed the overall study. Marjo Saastamoinen and Anna‐Liisa Laine provided sampling location information. Jonathan M. Chase helped with planning the local community sampling design. Lena Huovinen chose and validated the patches for sampling. Lena Huovinen and Anu Eskelinen collected the data. Lena Huovinen analysed the data and made the figures. Aleksi Räsänen validated connectivity measures and patch properties. Lena Huovinen and Anu Eskelinen wrote the manuscript and all authors contributed to editing the manuscript.

## Supporting information


**Data S1:** ele70257‐sup‐0001‐Supinfo.docx.

## Data Availability

The data that support the findings of this study are openly available in Dryad at https://doi.org/10.5061/dryad.2bvq83c2p and the code in Zenodo at https://doi.org/10.5281/zenodo.15321855.
