# Supplementary material for: Herbivory Modifies the Role of Spatial Processes in a Grassland Plant Metacommunity
Source: Ecol Lett. 2025 Nov 19;28(11):e70257. doi: 10.1111/ele.70257 (PMC12628290; doi:10.1111/ele.70257)
Supplement: Supplementary file 1 — Data S1: ele70257‐sup‐0001‐Supinfo.docx. [file ELE-28-0-s001.docx]

**Supplementary Information**

**Patch properties**

We only used grassland patches that had spatially continuous vegetation cover (i.e., were not excessively rocky), were treeless and not visibly disturbed by humans. Further, we did not include pastures with human-introduced sown species or transitioning grassland-forest vegetation. Of the 44 ungrazed patches, 21 had been previously grazed and later abandoned, while 23 had no grazing history during the past 30 years. However, these patches have likely been grazed a longer time ago, as otherwise they would not remain open/treeless. Further, most of the large currently ungrazed patches have been grazed at some point and were abandoned approximately 15-10 years ago (Table S2). Grazed patches had been continuously grazed for many decades and varied in the intensity of grazing, some being heavily and some only lightly grazed, and many were rotationally grazed (L. Huovinen and A. Eskelinen, personal observation). In general, our study landscape is characterized by a fine-grained mosaic of grazed and ungrazed patches that are often located within tens of meters from each other and occur on the same underlying habitat matrix. When choosing the patches for sampling, we excluded all extremely dry and low-productivity grasslands, irrespective of their grazing status; there were no significant differences in soil moisture and total soil nitrogen between grazed and ungrazed grasslands (Table S1). Further, there was no difference in the nearest distance, connectivity and area between grazed and ungrazed patches (Table S1). Although both large and small patches were more often grazed by cattle, they were also grazed by sheep and horses (data not shown).

Furthermore, we obtained 20 m spatial resolution CORINE Land Cover 2018 data from the Finnish Environment Institute (Syke). The data is based on supervised classification of satellite images and existing GIS data and is a national finer resolution version of the European-wide CORINE data (<https://ckan.ymparisto.fi/dataset/corine-maanpeite-2018>). We calculated the percentage cover of CORINE land cover types to check whether the 90 studied patches are within the same underlying matrix. We analysed all 90 patches used in the study with respect to water, forest, arable land (i.e., land suitable for growing crops; non-irrigated arable land, fruit trees and berry plantations, and arable land outside farming subsidies), and artificial surfaces (i.e., discontinuous urban fabric, commercial units, industrial units, road and rail networks and associated land, mineral extraction sites, summer cottages, sport and leisure areas, and golf courses). These analyses show that there is no relationship between the patch size to arable land (i.e., agricultural fields, orchards etc., which can be fertilized) either when considered in 1 km or 100 m radius around each patch (Tables S3 and S4). This relationship holds for area in general, and separately for grazed and ungrazed patches.

However, we found that area for ungrazed patches was correlated with proximity to artificial surfaces when measured in a 100 m radius around each patch (measured from the edge of each patch; Table S4). We are not sure why this correlation occurs, but it likely reflects that farmers tend to choose the currently grazed grasslands farther away from human influence where animals could face any kind of danger or disturbance by humans. It is also possible that the large, abandoned grasslands are more often located in the proximity of farms that have been abandoned 10-15 years ago (L. Huovinen & A. Eskelinen, personal observation). Most of the large currently ungrazed grasslands have been grazed more than 15 years ago. Artificial surfaces cover class includes, for example, golf courses but there are none this close to our patches in Åland Islands. This cover class also includes roads, however, Åland islands experiences relative low amount of traffic and there are no freeways or otherwise extensive road networks in the islands. The whole island system covers a land area of 1,527 km2, is inhabited by approximately 30 000 people (Statistics Finland 2023), and is mostly countryside. While there is no official data available on traffic rates in Åland, our personal observation is that traffic rates are very low outside the few main roads and none of our patches are directly alongside those main roads. Further, since our 1 m^2^ plots were always located in the centre of the patches (see Methods) and the large ungrazed patches are pretty large (5420-19196 m^2^; Table S2), they are inherently not right next to roadsides. We therefore do not believe this correlation affects our results.

**References**

European Environment Agency. (2019). CORINE Land Cover 2018 (raster 100 m), Europe, 6-yearly Version 2020_20u1, May 2020 (Version 20.01) [GeoTIFF]. European Environment Agency. <https://doi.org/10.2909/960998C1-1870-4E82-8051-6485205EBBAC>

Statistics Finland (2023). Statistical Yearbook of Finland 2023. *Helsinki: Statistics Finland*.

**Table S1**. Comparison of patch properties in grazed and ungrazed patches with linear regression models with total soil nitrogen, soil moisture, area, distance to the nearest patch and connectivity as response variables in separate models and herbivory as an explanatory variable. Total soil nitrogen (%N) was measured at plot level from air-dried samples and analysed with the Flash Smart CHNS/O (Thermo Scientific) element analyser at Oulu University. Soil moisture (VWC%; TDR 100, Spectrum Technologies, USA) was measured at plot level during plant community sampling. Both total soil nitrogen and moisture were averaged per habitat patch for the models.

| **Response variable** | Explanatory variable  Herbivory (grazed/ungrazed)  Linear regression model fit by REML  F-test and p-value | | | |
| --- | --- | --- | --- | --- |
|  | Coef. | Std. Error | F | P |
| Total soil nitrogen (%N) | 0.02 | 0.04 | 0.1 | 0.7261 |
| Soil moisture (%VWC) | 2.09 | 1.22 | 2.9 | 0.0912 |
| Area (m^2^) | -848.9 | 1043.5 | 0.7 | 0.4181 |
| Distance to the nearest patch (m) | -4.5 | 17.40 | 0.1 | 0.796 |
| Connectivity | -2959498 | 3783593 | 0.6 | 0.4362 |

**Table S2:** Area and grazing history of the largest ungrazed patches (ca. 33% of the largest ungrazed patches), showing that most large ungrazed patches have been grazed more than 10-15 years ago, after which grazing was abandoned.

| Patch nr | ln (area) | Area (m^2^) | Grazing history |
| --- | --- | --- | --- |
| 4945 | 9.86 | 19196 | grazed until 2010 |
| 1097 | 9.65 | 15504 | no grazing record |
| 1602 | 9.56 | 14232 | grazed until 2015 |
| 703 | 9.36 | 11596 | grazed until 2013 |
| 9219 | 9.30 | 10975 | no grazing record |
| 511 | 9.28 | 10748 | grazed until 2014 |
| 689 | 9.23 | 10181 | grazed until 2013 |
| 483 | 9.16 | 9526 | no grazing record |
| 2101 | 9.02 | 8295 | no grazing record |
| 1067 | 8.86 | 7074 | grazed until 2015 |
| 4936 | 8.85 | 6986 | grazed until 2010 |
| 601 | 8.75 | 6326 | grazed until 2014 |
| 462 | 8.73 | 6182 | no grazing record |
| 509 | 8.70 | 6025 | grazed until 2014 |
| 1513 | 8.60 | 5420 | grazed until 2019 |

**Table S3:** Pearson correlation of percentage land cover type within 1000 m of each patch and habitat area in all patches and separately in grazed and ungrazed patches. We grouped level three CORINE classes into the following categories. Artificial surface: discontinuous urban fabric, commercial units, industrial units, road and rail networks and associated land, mineral extraction sites, summer cottages, sport and leisure areas, and golf courses. Arable land and plantations: non-irrigated arable land, fruit trees and berry plantations, and arable land outside farming subsidies. Forest: agro-forestry areas, broad-leaved forest on mineral soil, broad-leaved forest on peatland, coniferous forest on mineral soil, coniferous forest on peatland, coniferous forest on rocky soil, mixed forest on mineral soil, mixed forest on peatland, mixed forest on rocky soil. Water: water courses, water bodies and sea and ocean.

| Spatial variable | Artificial surface (%) | | Arable land & plantations (%) | | Forest (%) | | Water (%) | |
| --- | --- | --- | --- | --- | --- | --- | --- | --- |
|  | r | p | r | p | r | p | r | p |
| All patches | | | | | | | | |
| Area (m^2^) | 0.14 | 0.1991 | -0.07 | 0.5022 | 0.12 | 0.2476 | -0.01 | 0.8926 |
| Ln area (m^2^) | 0.06 | 0.5739 | -0.02 | 0.8631 | 0.09 | 0.4057 | -0.06 | 0.5657 |
| Grazed only | | | | | | | | |
| Area (m^2^) | 0.20 | 0.1936 | -0.09 | 0.5662 | 0.24 | 0.1144 | -0.04 | 0.7690 |
| Ln area (m^2^) | 0.02 | 0.9164 | -0.10 | 0.5054 | 0.21 | 0.1575 | -0.07 | 0.6430 |
| Ungrazed only | | | | | | | | |
| Area (m^2^) | 0.02 | 0.9110 | -0.07 | 0.6575 | 0.03 | 0.8630 | 0.03 | 0.8233 |
| Ln area (m^2^) | 0.11 | 0.4625 | 0.04 | 0.8066 | 0.03 | 0.8329 | -0.06 | 0.6967 |

**Table S4:** Pearson correlation of percentage land cover type within 100 m of each patch and habitat area in all patches and separately in grazed and ungrazed patches. We grouped level three CORINE classes into the following categories. Artificial surface: discontinuous urban fabric, commercial units, industrial units, road and rail networks and associated land, summer cottages. Pastures: pastures, natural pastures. Arable land and plantations: non-irrigated arable land, fruit trees and berry plantations, and arable land outside farming subsidies. Forest: agro-forestry areas, broad-leaved forest on mineral soil, broad-leaved forest on peatland, coniferous forest on mineral soil, coniferous forest on peatland, coniferous forest on rocky soil, mixed forest on mineral soil, mixed forest on peatland, mixed forest on rocky soil. Water: water courses, water bodies and sea and ocean.

| Spatial variable | Artificial surface | | Arable land & plantations | | Forest | | Water | |
| --- | --- | --- | --- | --- | --- | --- | --- | --- |
|  | r | p | r | p | r | p | r | p |
| All patches | | | | | | | | |
| Area (m^2^) | 0.07 | 0.5396 | -0.05 | 0.6263 | -0.08 | 0.4748 | 0.06 | 0.5517 |
| Ln area (m^2^) | 0.09 | 0.4089 | -0.05 | 0.6485 | -0.09 | 0.4094 | 0.07 | 0.4962 |
| Grazed only | | | | | | | | |
| Area (m^2^) | -0.10 | 0.5005 | 0.02 | 0.8840 | 0.10 | 0.5146 | -0.08 | 0.5772 |
| Ln area (m^2^) | -0.17 | 0.2731 | 0.08 | 0.5939 | 0.03 | 0.8612 | -0.08 | 0.6051 |
| Ungrazed only | | | | | | | | |
| Area (m^2^) | 0.26 | 0.0861 | -0.10 | 0.5015 | -0.23 | 0.1419 | 0.23 | 0.1407 |
| Ln area (m^2^) | 0.30 | **0.0448** | -0.12 | 0.4421 | -0.13 | 0.3968 | 0.19 | 0.2181 |

**Results**


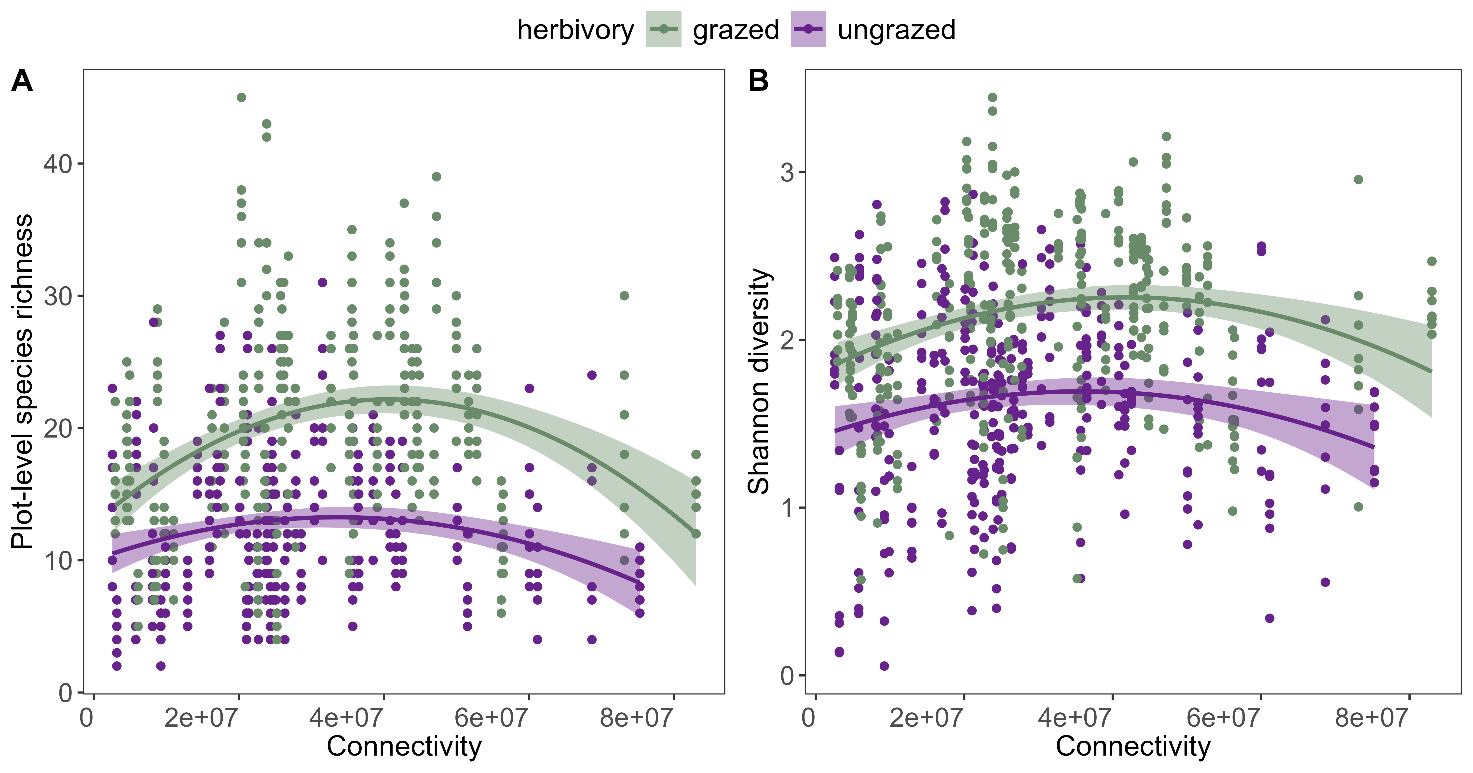


**Figure S1**. (a) The relationship between plant species richness and connectivity (R^2^ _conditional_ = 0.81, p=0.0051) and (b) the relationship between Shannon diversity and connectivity (R^2^ _conditional_ = 0.68, p=0.0475) in fixed sized 1 m^2^ plots (eight replicates per patch).


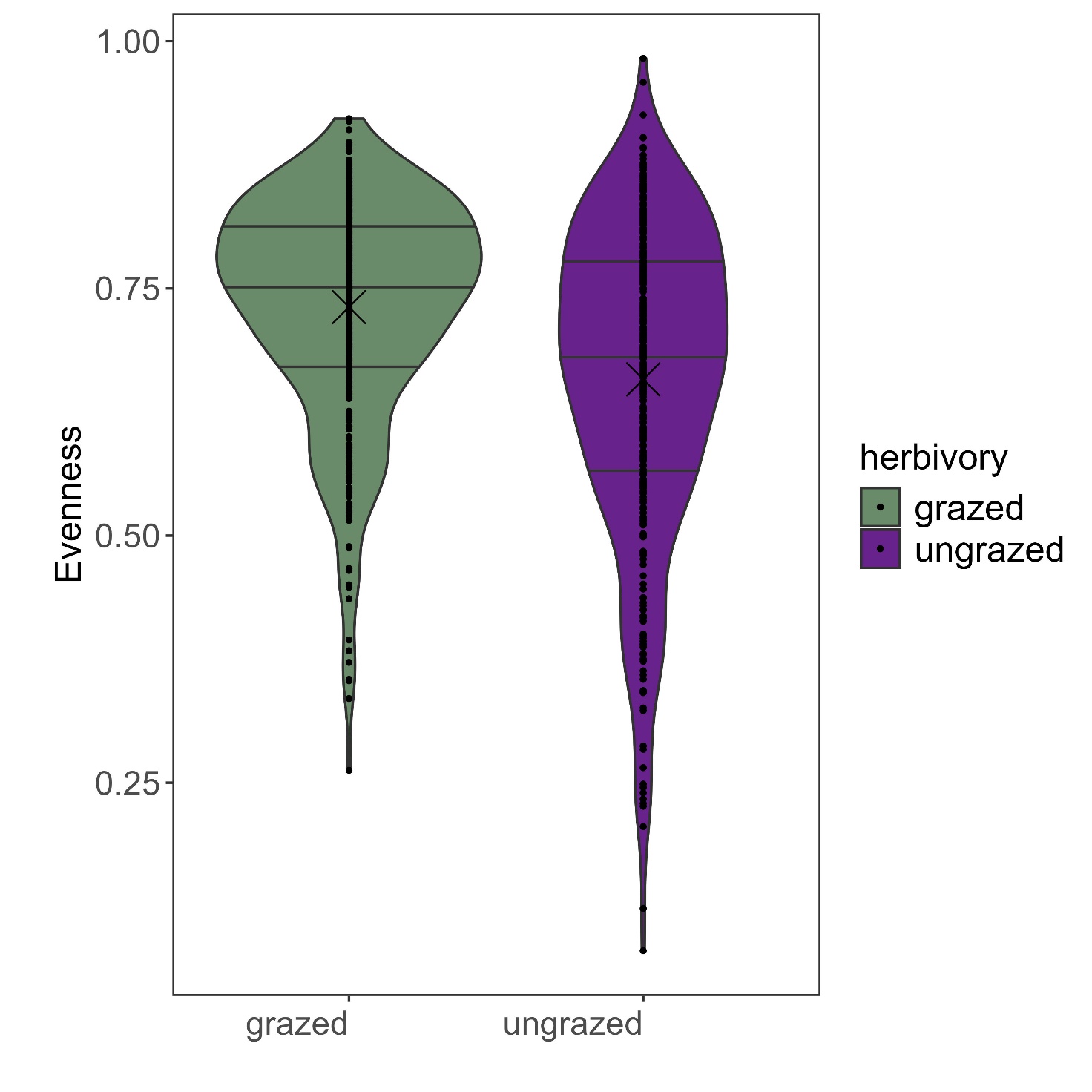


**Figure S2**. Pielou´s Evenness (R^2^ _conditional_ = 0.36, p=0.0001) in grazed and ungrazed fixed sized 1 m^2^ plots (eight replicates per patch).

**Table S5**. Results of mixed effects models where plot level richness, Shannon diversity and evenness were response variables and herbivory, area, distance to the nearest patch (ND) and its quadratic term (to test unimodal relationship), and the interactions between area and herbivory and distance to the nearest patch and herbivory. Patch was used as a random variable. We used a Generalised Least Squares model fitted for spatial autocorrelation to test how the same predictor variables as above were related to whole grassland level richness. P-values ≤ 0.05 are boldfaced. We used ln transformation for area in all models.

|  | **Whole patch richness**  DF= 1,81  Generalised Least Squares model fitted for spatial autocorrelation  F-test for p-value  R^2^_nagelkerke_= 0.43 | | | | **Plot level richness**  Linear mixed-effects model fit by REML  F-test for p-value  DF = 1,81  Random effects:  5.59, residual 3.46  marginal R^2^= 0.32, conditional R^2^= 0.81 | | | | **Plot level Shannon**  Linear mixed-effects model fit by REML  F-test for p-value  DF =1,81  Random effects:  0.39, residual 0.35  marginal R^2^= 0.26, conditional R^2^= 0.68 | | | | **Plot level Evenness**  DF = 1,81  Linear mixed-effects model fit by REML  F-test for p-value  Random effects:  0.08, residual 0.12  marginal R^2^= 0.09, conditional R^2^= 0.36 | | | |
| --- | --- | --- | --- | --- | --- | --- | --- | --- | --- | --- | --- | --- | --- | --- | --- | --- |
| **Predictor variables** | Coef. | Std. Error | F | P | Coef. | Std. Error | F | P | Coef. | Std. Error | F | P | Coef. | Std. Error | F | P |
| ND | 0.08 | 0.11 | 0.5 | 0.4659 | 0.15 | 0.05 | 0.01 | 0.9036 | < 0.01 | < 0.01 | <0.1 | 0.9921 | < 0.01 | < 0.01 | 0.2 | 0.6960 |
| Herbivory | 32.33 | 20.75 | 34.8 | **<0.0001** | 18.6 | 9.12 | 38.0 | **<0.0001** | 1.25 | 0.66 | 36.6 | **<0.0001** | 0.16 | 0.14 | 17.2 | **0.0001** |
| ND^2 | <-0.01 | < 0.01 | 1.0 | 0.3186 | <-0.01 | < 0.01 | 8.3 | **0.0050** | < -0.01 | <0.01 | 7.4 | **0.0080** | <-0.01 | < 0.01 | 3.4 | 0.0676 |
| Area | 9.21 | 1.78 | 27.9 | **<0.0001** | 1.10 | 0.81 | 0.4 | 0.5137 | 0.10 | 0.06 | 0.05 | 0.8298 | 0.02 | 0.01 | 0.3 | 0.6092 |
| ND × herbivory | -0.11 | 0.15 | 0.8 | 0.3763 | -0.1 | 0.07 | 0.6 | 0.4279 | <-0.01 | < 0.01 | 1.0 | 0.3255 | <-0.01 | < 0.01 | 0.4 | 0.5194 |
| ND^2 × herbivory | < 0.01 | < 0.01 | 0.7 | 0.4095 | < 0.01 | < 0.01 | 2.6 | 0.1094 | < 0.01 | < 0.01 | 0.9 | 0.3426 | <-0.01 | < 0.01 | 0.02 | 0.8823 |
| Area × herbivory | -5.10 | 2.50 | 4.2 | **0.0441** | -2.59 | 1.10 | 5.6 | **0.0209** | -0.19 | 0.08 | 5.6 | **0.0201** | -0.03 | 0.02 | 2.6 | 0.1098 |

**Table S6**. Results of mixed effects models where plot level richness, Shannon diversity and evenness were response variables and herbivory, area, a second-order polynomial function of connectivity, and the interactions between area and herbivory and connectivity and herbivory. Patch was used as a random variable. We used a Generalised Least Squares model fitted for spatial autocorrelation to test how the same predictor variables as above were related to whole grassland level richness. P-values ≤ 0.05 are boldfaced**.** We used ln transformation for area in all models.

|  | **Whole patch richness**  Generalised Least Squares model fitted for spatial autocorrelation  F-test for p-value  DF= 1,82  R^2^_nagelkerke_= 0.42 | | | | **plot level Richness**  DF = 1,84  Linear mixed-effects model fit by REML  F-test for p-value  Random effects: 5.62, residual 3.48  marginal R^2^= 0.31, conditional R^2^= 0.81 | | | | **plot level Shannon**  DF =1,84  Linear mixed-effects model fit by REML  F-test for p-value  Random effects: 0.41, residual 0.35  marginal R^2^= 0.23, conditional R^2^= 0.67 | | | | **plot level Evenness**  DF = 1,84  Linear mixed-effects model fit by REML  F-test for p-value  Random effects: 0.08, residual 0.12  marginal R^2^= 0.08, conditional R^2^= 0.37 | | | |
| --- | --- | --- | --- | --- | --- | --- | --- | --- | --- | --- | --- | --- | --- | --- | --- | --- |
| **Predictor variables** | Coef. | Std. Error | F | P | Coef. | Std. Error | F | P | Coefficients | Std. Error | F | P | Coefficients | Std. Error | F | P |
| poly(Connectivity,2) | -24.43 | 17.83 | 0.9 | 0.4218 | -60.61 | 21.16 | 5.3 | **0.0051** | -2.70 | 1.56 | 3.1 | **0.0475** | -0.08 | 0.32 | 0.7 | 0.5073 |
| Herbivory | 32.02 | 21.24 | 25.8 | **<0.0001** | 10.90 | 8.93 | 36.2 | **<0.0001** | 0.76 | 0.66 | 32.5 | **<0.0001** | 0.10 | 0.13 | 15.2 | **0.0002** |
| Area | 9.2 | 1.8 | 26.3 | **<0.0001** | 1.10 | 0.81 | <0.1 | 0.9399 | 0.10 | 0.06 | 0.1 | 0.7129 | 0.02 | 0.01 | 0.9 | 0.3371 |
| poly(Connectivity,2)x herbivory | 17.70 | 28.42 | 0.8 | 0.4539 | 38.62 | 33.80 | 1.0 | 0.3798 | 0.81 | 2.49 | 0.2 | 0.8185 | -0.21 | 0.52 | 0.2 | 0.8512 |
| Area × herbivory | -5.68 | 2.58 | 4.8 | **0.0306** | -2.26 | 1.10 | 4.3 | **0.0420** | -0.16 | 0.08 | 3.8 | **0.0547** | -0.02 | 0.02 | 1.5 | 0.2206 |

**Table S7.** Results of mixed effects models where CWM traits were response variables and herbivory, area, distance to the nearest patch (ND), and the interactions between area and herbivory and distance to the nearest patch and herbivory were fixed predictor variables. Patch was used as a random variable. P-values ≤ 0.05 are indicated in bold. Height and dispersal ability were square root transformed. We used ln transformation for the area in all models.

|  | **CWM height**  Linear mixed-effects model fit by REML  F-test for p-value  DF = 1,83  Random effect: 0.72, residual 0.61  marginal R^2^= 0.22, conditional R^2^= 0.68 | | | | **CWM C:N**  Linear mixed-effects model fit by REML  F-test for p-value  DF = 1,83  Random effect: 3.00, residual 3.19  marginal R^2^= 0.15, conditional R^2^= 0.55 | | | | **CWM WUE**  Linear mixed-effects model fit by REML  F-test for p-value  DF = 1,83  Random effect: 0.27, residual 0.35,  marginal R^2^= 0.09, conditional R^2^= 0.43 | | | | **CWM dispersal ability**  Linear mixed-effects model fit by REML  F-test for p-value  DF = 1,83  Random effect: 0.11, residual 0.11  marginal R^2^= 0.07, conditional R^2^= 0.51 | | | |
| --- | --- | --- | --- | --- | --- | --- | --- | --- | --- | --- | --- | --- | --- | --- | --- | --- |
| **Predictor variables** | Coef. | Std. Error | F | P | Coef. | Std. Error | F | P | Coef. | Std. Error | F | P | Coef. | Std. Error | F | P |
| ND | < 0.01 | < 0.01 | <0.1 | 0.9874 | < - 0.01 | < 0.01 | 2.8 | 0.0959 | < 0.01 | < 0.01 | 0.1 | 0.8185 | < 0.01 | < 0.01 | 2.4 | 0.1260 |
| Herbivory | -0.17 | 1.17 | 34.4 | **<0.0001** | -6.08 | 4.98 | 25.3 | **<0.0001** | 0.91 | 0.47 | 15.8 | **0.0002** | 0.42 | 0.18 | 0.1 | 0.7180 |
| Area | 0.04 | 0.11 | 3.2 | 0.0778 | -0.34 | 0.45 | 0.2 | 0.6189 | < 0.01 | 0.04 | 1.6 | 0.2091 | < 0.01 | 0.02 | 4.0 | **0.0490** |
| ND × herbivory | < - 0.01 | < 0.01 | < 0.1 | 0.9363 | < - 0.01 | 0.01 | 0.1 | 0.7522 | < - 0.01 | < 0.01 | 0.1 | 0.7911 | < 0.01 | < 0.01 | 0.7 | 0.3892 |
| Area × herbivory | 0.15 | 0.14 | 1.1 | 0.3078 | 0.37 | 0.61 | 0.4 | 0.5516 | < - 0.08 | 0.06 | 2.0 | 0.1638 | -0.06 | 0.02 | 6.7 | **0.0113** |

**Table S8.** Results of mixed effects models where CWM traits were response variables and herbivory, area, connectivity, and the interactions between area and herbivory and connectivity and herbivory were fixed predictor variables. Patch was used as a random variable. P-values ≤ 0.05 are indicated in bold. Height and dispersal ability were square root transformed. We used ln transformation for the area in all models.

|  | **CWM height**  Linear mixed-effects model fit by REML  F-test for p-value  DF = 1,84  Random effect: 0.72, residual 0.61  marginal R^2^= 0.21, conditional R^2^= 0.68 | | | | **CWM C:N**  Linear mixed-effects model fit by REML  F-test for p-value  DF = 1,84  Random effect: 3.1, residual 3.2  marginal R^2^= 0.13, conditional R^2^= 0.55 | | | | **CWM WUE**  Linear mixed-effects model fit by REML  F-test for p-value  DF = 1,84  Random effect: 0.27, residual 0.35  marginal R^2^= 0.09, conditional R^2^= 0.43 | | | | **CWM dispersal ability**  Linear mixed-effects model fit by REML  F-test for p-value  DF = 1,84  Random effect: 0.11, residual 0.11  marginal R^2^= 0.07, conditional R^2^= 0.52 | | | |
| --- | --- | --- | --- | --- | --- | --- | --- | --- | --- | --- | --- | --- | --- | --- | --- | --- |
| **Predictor variables** | Coef. | Std. Error | F | P | Coef. | Std. Error | F | P | Coef. | Std. Error | F | P | Coef. | Std. Error | F | P |
| Connectivity | <0.01 | <0.01 | 2.2 | 0.1447 | <0.01 | <0.01 | 0.8 | 0.3844 | <0.01 | <0.01 | 1.4 | 0.2342 | <0.01 | <0.01 | 0.2 | 0.6634 |
| Herbivory | 0.10 | 1.18 | 33.2 | **<0.0001** | -6.41 | 5.14 | 22.2 | **<0.0001** | 1.03 | 0.47 | 13.7 | **0.0004** | 0.49 | 0.18 | <0.1 | 0.9548 |
| Area | 0.05 | 0.11 | 3.1 | 0.0827 | -0.36 | 0.46 | 0.5 | 0.4610 | 0.01 | 0.04 | 2.0 | 0.1584 | 0.01 | 0.02 | 3.6 | 0.0591 |
| Connectivity × herbivory | <0.01 | <0.01 | 0.5 | 0.4895 | <0.01 | <0.01 | 0.9 | 0.3491 | <0.01 | <0.01 | 0.7 | 0.3926 | <0.01 | <0.01 | 2.9 | 0.0912 |
| Area × herbivory | 0.13 | 0.14 | 0.8 | 0.3687 | 0.25 | 0.61 | 0.2 | 0.6832 | -0.09 | 0.06 | 2.5 | 0.1190 | -0.05 | 0.02 | 5.8 | **0.0179** |

**Detailed description of trait measurement methods.** For plant height and SLA, we measured 5-10 naturally occurring mature plant individuals that did not show any signs of disease or herbivory. The measurements were always done from plant individuals from several separate grasslands. Plant height was measured from the ground to the highest photosynthetic tissue without uplifting the plant. For SLA, we collected mature leaves including petioles and stipules, placed them in Ziploc bags with wet paper towels, and stored in cool box/fridge until analysis. The leaves were weighed and scanned (Canon, CanoScan Lide 400). We used ImageJ to analyse the leaf area scans.

For the chemical analysis, we collected ca. 2 g of fresh leaf material (no stems were included). A subset of the leaf-material was first air-dried and then freeze-dried (Edwards Vacuum) for the tannin analysis. Another subset was oven-dried at 40 C ° for at least 2 days for the C:N and WUE analyses. The samples for all chemical analyses were ground in a laboratory mixer mill (RETSCH MM301). For C:N, after milling, 7-8 mg of the plant powder was measured into tin cups and then analysed with the Flash Smart CHNS/O (Thermo Scientific Inc, Milan, Italy) element analyser at Oulu University. Condensed tannins were analysed by the natural chemistry research group at the University of Turku using an HCl-butanol assay (Shay et al. 2017). WUE was analysed at the Finnish Museum of Natural History (LUOMUS) as the ratio of 13C/12C isotopes compared to the VPDB standard.

**References**

Shay, P.-E., Trofymow, J. A., & Constabel, C. P. (2017). An improved butanol-HCl assay for quantification of water-soluble, acetone:methanol-soluble, and insoluble proanthocyanidins (condensed tannins). *Plant Methods*, 13(1), 63.
